# Tumor-associated fibroblast-conditioned medium induces CDDP resistance in HNSCC cells

**DOI:** 10.18632/oncotarget.6210

**Published:** 2015-10-20

**Authors:** Teresa Bernadette Steinbichler, Veronika Metzler, Christian Pritz, Herbert Riechelmann, Jozsef Dudas

**Affiliations:** ^1^ Department of Otorhinolaryngology, Medical University of Innsbruck, Innsbruck, Austria

**Keywords:** epithelial to mesenchymal transition, chemoresistance, cisplatin, cancer-associated fibroblasts, TGF-β1

## Abstract

**Objective:**

EMT (epithelial to mesenchymal transition) contributes to tumor progression and metastasis. We aimed to investigate the effects of EMT on CDDP resistance in HNSCC (head and neck squamous cell carcinoma)-cells.

**Methods:**

EMT was induced using conditioned medium from a tumor cell/fibroblast co-culture. HNSCC cells were alternatively treated with TGF-β1. The response to CDDP was evaluated with viability and clonogenic assays.

**Results:**

Treatment of SCC-25/Detroit 562 cells with conditioned medium increased viability of the tumor cells. Moreover, it doubled the IC_50_ of CDDP of SCC-25 cells from 6.2 μM to 13.1 μM (*p* < 0.001). The IC_50_ of CDDP of Detroit 562 cells was increased following treatment with conditioned medium from 13.1 μM to 26.8 μM (*p* < 0.01). Colony forming ability after treatment with 5 or 10 μM CDDP was significantly higher in HNSCC cells treated with co-culture conditioned medium than in controls (*p* < 0.05). Treatment with TGF-β1 had no effect on the IC_50_ of CDDP (*p* > 0.1).

**Conclusions:**

Cell free medium from a co-culture was able to induce EMT in HNSCC cells. Co-culture treated HNSCC cells revealed increased viability and were less sensitive to CDDP treatment. TGF-β1 also induced a mesenchymal phenotype, but did not alter resistance to CDDP in HNSCC cells.

## INTRODUCTION

Most studies on chemotherapy resistance of tumor cells focus on genetic or phenotypic alterations of the cancer cell itself. However, there is growing evidence that the stroma of solid tumors interacts with cancer cells. Tumor-stroma interaction is considered a significant determinant of disease progression and metastasis [1, 2]. Stromal fibroblasts induce epithelial to mesenchymal transition (EMT) in head and neck squamous cell carcinoma (HNSCC) tumor cells [3]. EMT is a reversible cellular process mainly induced by paracrine secretion of small molecules by tumor-associated fibroblasts [4]. Among these small molecules, transforming growth factor-β1 (TGF-β1) is thought to be one of the most relevant mediators [5, 6]. TGF-β1 interacts with TGF-β1 two type I and two type II transmembrane kinase receptors. These receptors activate Smad 2 and 3 signaling pathways, which form a complex with Smad 4, leading to the expression of EMT-activating transcriptional factors such as Snail/Slug [7]. Two major hallmarks of EMT in epithelial tumor cells are increased expression of the intermediate filament vimentin and decreased expression of E-cadherin [3]. We recently reported that EMT in HNSCC-cell lines results in enhanced cell proliferation [8]. In this study, we analyze if EMT increases CDDP (Cisplatin) resistance in two HNSCC cell lines.

SCC-25 cells were orignially isolated from the primary tumor of a patient with tongue carcinoma [9]. SCC-25 cells form tumors in SCID mice but not in athymic nude mice suggesting less aggressive behavior. Moreover, SCC-25 induced tumors do not develop regional or distant metastasis in mouse models [10]. In contrast, Detroit 562 cells grow tumors and develop regional and lung metastases when injected in nude mice [11]. Detroit 562 was isolated from the malignant pleural effusion of a patient with pharyngeal carcinoma [12, 13].

Previously, we had induced EMT by co-cultivation of SCC-25 or Detroit 562 cells and fibroblasts [1]. The resulting co-culture conditioned medium containing EMT-promoting factors was used in this study to induce EMT in pure SCC-25 or Detroit 562 cells. In a second experimental arm, tumor cells were treated with TGF-β1. In a third experimental arm tumor cells were treated with co-culture conditioned medium and a neutralizing dose of anti-TGF-β antibody. SCC-25 and Detroit 562 cells treated with standard cell culture medium served as control in a fourth experimental arm. Changes of vimentin and E-cadherin gene expression and protein synthesis, which were evaluated by quantitative real-time polymerase chain reaction (PCR) and by western blotting were used to confirm EMT. Cell viability was determined with MTT assays and colony-forming ability was investigated using clonogenic assays [14]. Response to CDDP was assessed by cell viability and Clonogenic assays.

## RESULTS

### Co-culture conditioned medium and TGF-β1 induced EMT in SCC-25 cells

**Figure 1:**
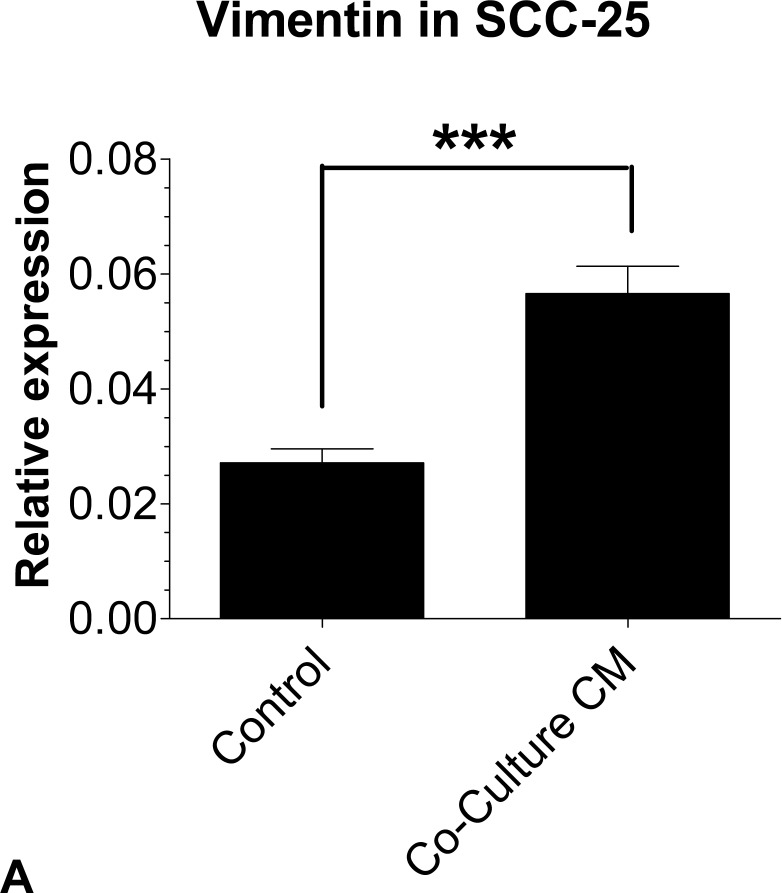

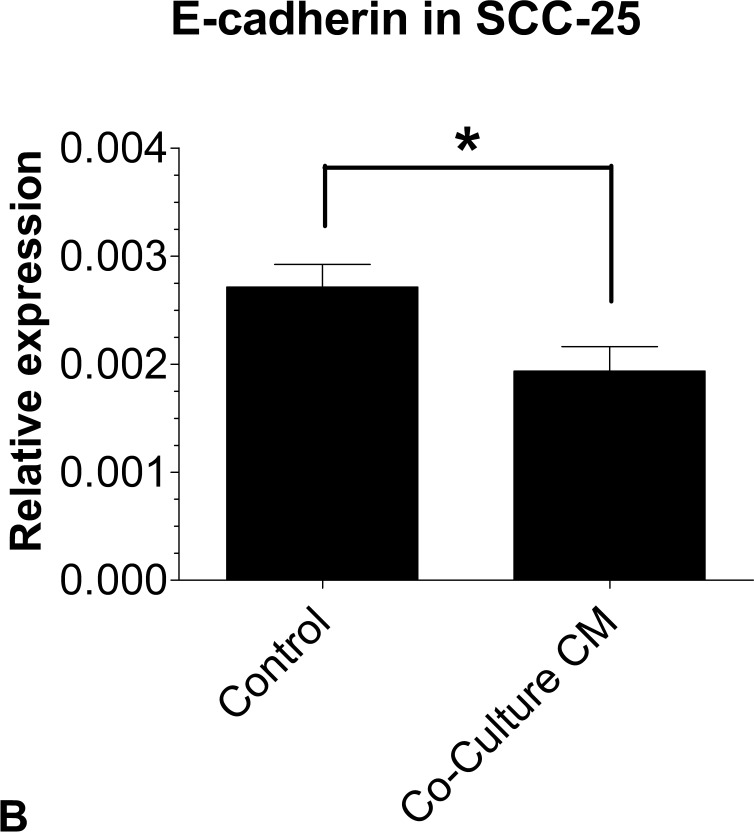

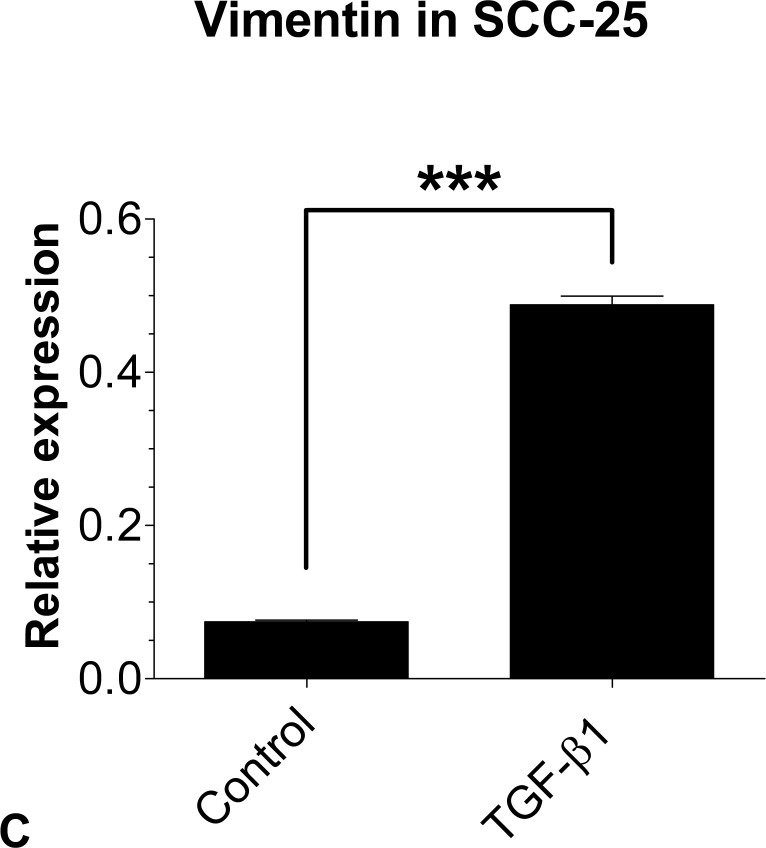

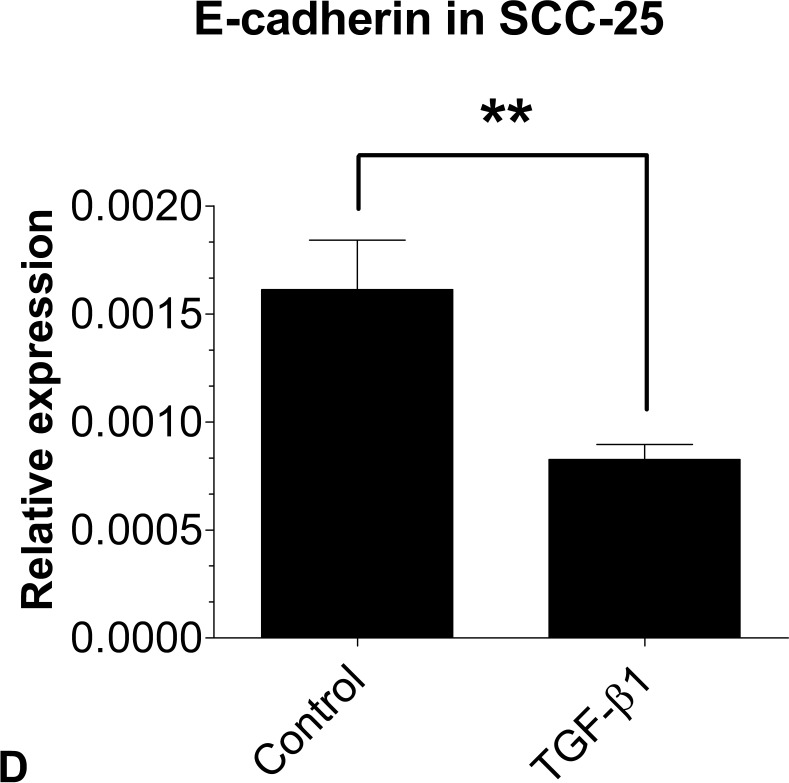
EMT-related gene expression in SCC-25 using real time- PCR: The mRNA expressions of the EMT markers vimentin and E-cadherin in SCC-25 cells treated with co-culture conditioned medium (A, B) or 0.9 ng/ml TGF-β1 (C, D) were quantified relative to SCC-25 control cells Co-culture conditioned medium treated SCC-25 cells show a significant increase of vimentin **A.** mRNA expression, while E-cadherin **B.** mRNA expression was significantly decreased. The treatment with 0.9 ng/ml TGF-β1 led to the same effect to an even greater extent with a highly significant upregulation of vimentin **C.** and downregulation of E-cadherin **D.** in SCC-25 cells. Experiments were performed in three replicates with three runs each. *p < 0.05, **p < 0.01, ***p < 0.001.

SCC-25 cells were treated with standard medium (control), co-culture conditioned medium or 1 ng/ml TGF-β1 for 3 days. EMT-related gene expression was subsequently measured using real-time PCR and compared with β-actin expression (Figure 1). Relative gene expression of the mesenchymal marker vimentin was higher in SCC-25 cells treated with co-culture conditioned medium (56.6±22.4×10^−3^) than in control cells (27.1±10.3×10^−3^; *p* < 0.001). In contrast, E-cadherin gene expression was down regulated following treatment with co-culture conditioned medium (1.9±1.1×10^−3^) compared to controls (2.7±0.9×10^−3^; *p* < 0.02). EMT-like gene expression changes were also observed in SCC-25 cells treated with 1 ng/ml TGF-β1. TGF-β1 treated cells revealed significantly higher vimentin gene expression (488.1±48 × 10^3^) than control cells (74.4±10.1× 10^−3^; *p* < 0.001) and lower E-cadherin gene expression levels (0.8±0.3× 10^−3^) compared to controls (1.6±0.9× 10^−3^; *p* < 0.01). At protein level both, co-culture conditioned medium and 1 ng/ml TGF-β1, increased a 46 kD vimentin band. E-cadherin showed a marginal decrease after treatment with co-culture conditioned medium and 1 ng/ml TGF-β1 in SCC-25 cells. Moreover, cell viability of SCC-25 cells treated with co-culture conditioned medium was higher (1.25±0.11) than in untreated controls (1.09±0.23; *p* < 0.01). In contrast, treatment with TGF-β1 decreased cell viability (0.69±0.007; *p* < 0.001) compared to controls treated with albumin-containing medium (Figure 3A).

A neutralizing assay with anti-TGF-β antibody did not reduce the influence of co-culture conditioned medium on cell viability (1.45±0.03; *p* > 0.1). The number of colonies in clonogenic assays did not differ between standard-medium (3116±654) and co-culture conditioned medium (1805±131; *p* > 0.5). Furthermore treatment with TGF-β1 (2547±80) or co-culture conditioned medium and anti-TGF-β (1496±119) antibody had no influence on clonogenity of SCC-25 cells (*p* > 0.1) (Table 1).

**Table 1:** Co-culture conditioned Medium and TGF-β1 induced EMT in SCC-25 cells and did not influence the phenotype of Detroit 562 cells

Cell type	Test	conditioned Medium	TGF-β1
**SCC-25**	Vimentin PCR (RNA)	upregulation***	upregulation***
	E-cadherin PCR (RNA)	downregulation*	downregulation**
	vimentin Western Blot (protein)	upregulation (46kD)	upregulation (46kD)
	E-cadherin Western blot (protein)	downregulation	downregulation
	viability	increase**	decrease***
	clonogenity	no change	no change
**Detroit 562**	Vimentin PCR (RNA)	no change	upregulation***
	E-cadherin PCR (RNA)	no change	no change
	Vimentin Western blot (protein)	no change	no change
	E-cadherin Western blot (protein)	downregulation	downregulation
	viability	increase***	no change
	clonogenity	no change	no change

* p < 0.05

** p < 0.01

*** p < 0.001

### Conditioned medium did not induce an EMT like phenotype in detroit 562 cells

**Figure 2:**
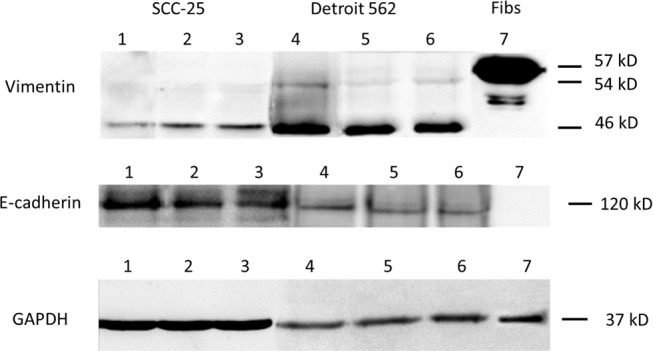
EMT-related protein synthesis in SCC-25 and Detroit 562 cells Western blots of (supernatants/cell homgenisates), representative sample of three replicates. Lane 1: SCC-25 cells in albumin-containing medium (control), Lane 2: SCC-25 treated with co-culture conditioned medium, Lane 3: SCC-25 treated with 1 ng/ml TGF-β1, Lane 4: Detroit 562 in albumin-containing medium (control), Lane 5: Detroit 562 treated with co-culture conditioned medium, Lane 6: Detroit 562 treated with 1 ng/ml TGF-β1, Lane 7: human gingival fibroblasts. Vimentin was detected in 57, 54 and 46 kD bands. In SCC-25 cells, co-culture conditioned medium and 1 ng/ml TGFβ1 increased the 54 and 46 KD bands. There was a high constitutive expression of 46 kD vimentin in Detroit 562 cells without any treatment. The treatment with co-culture conditioned medium did not increase both vimentin bands. The positive control HGF fibroblasts showed a strong band at 57 kD. E-cadherin was detected at 120 kD. SCC-25 cells at control conditions showed a strong 120 kD band, which was reduced after treatment with co-culture conditioned medium or with 1 ng/ml TGF-β1. Detroit 562 cells showed a light band at 120 kD, which was marginally reduced after treatment with co-culture conditioned medium or with 1 ng/ml TGF-β1 The control HG-fibroblasts did not not express protein at 120 kD with the vimentin antibody. Loading control was done using anti-GAPDH antibody.

Treatment with co-culture conditioned medium did not induce significant phenotypic changes in Detroit 562 cells. Vimentin expression (0.59±0.04×10^−^3 *vs*. 0.58±0.2×10^−3^, *p* > 0.1) and E-cadherin expression (3.6±2.6×10^−3^ *vs*. 4.6±0.2×10^−3^, *p* > 0.1) did not show significant changes after treatment with co-culture conditioned medium. Treatment with TGF-β1 induced EMT-like phenotypic changes in Detroit 562 cells. TGF-β1 increased vimentin expression about ten fold (5.6±1.4×10^−3^ *vs*. 0.58±0.2×10^−3^, *p* < 0.0001), but failed to reduce E-cadherin expression (17.5±1×10^−3^, *p* > 0.1). At protein level, both, conditioned medium and 1 ng/ml TGF-β1 did not induce changes in vimentin expression (46 kD band) (Figure 2). E-cadherin showed a marginal decrease after treatment with co-culture conditioned medium and 1 ng/ml TGF-β1. Viability increased after treatment with co-culture conditioned medium (2.1±0.04) compared to controls (1.9±0.02, *p* = 0.001), whereas TGF-β1 had no influence on cell viability (1.9±0.005, *p* > 0.5). A neutralizing assay with anti-TGF-β antibody did not reduce the effect of co-culture conditioned medium on cell viability (2.2±0.01, *p* > 0.05). Treatment with co-culture conditioned medium, TGF-β1 and co-culture conditioned medium/anti-TGF-β antibody had no effect on clonogenity in Detroit 562 cells (standard medium 948±153, CM: 983±38, TGF-β1 876±160; anti-TGF-β: 1000±18; *p* > 0.1) (Table 1).

### Treatment with co-culture conditioned medium increased CDDP resistance of SCC-25 cells

**Figure 3:**
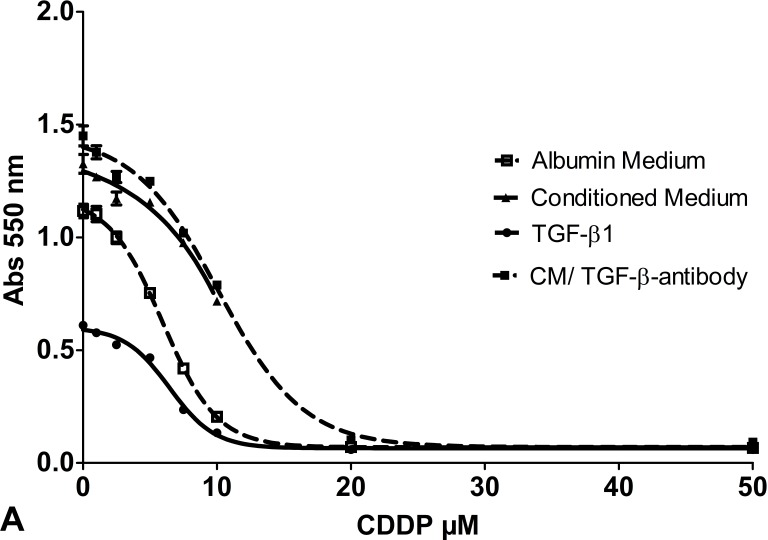

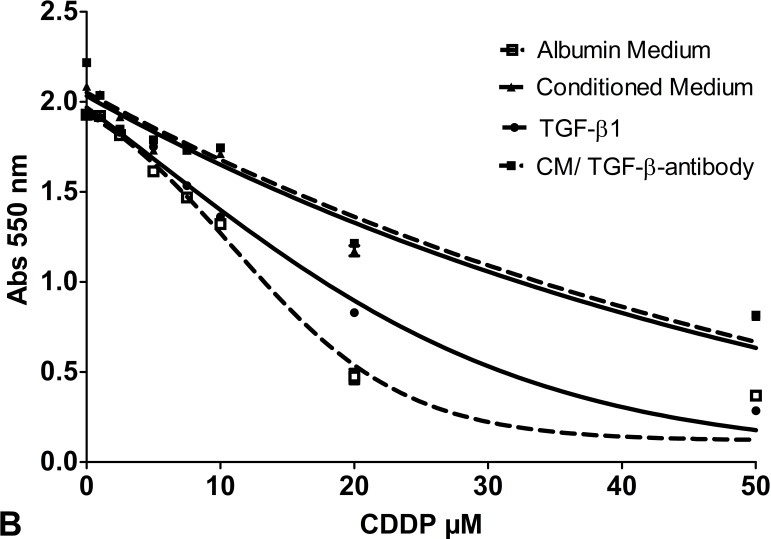
Cell viability assay: Cell viability of SCC-25 cells **A.** or Detroit 562 cells fat as well **B.** exposed to increasing doses of CDDP (0- 50 μM) following treatment with albumin containing medium (control; dotted line with white squares), co-culture conditioned medium (solid line with triangles), medium supplemented with TGF-β1 0.9 ng/ml (dotted line with spheres) and co-culture conditioned medium plus anti TGF-β antibody (1.5 μg/ml) (solid line with black squares). Four parameter nonlinear logistic regression model, whiskers indicate standard error of the mean (SEM).

**Figure 4:**
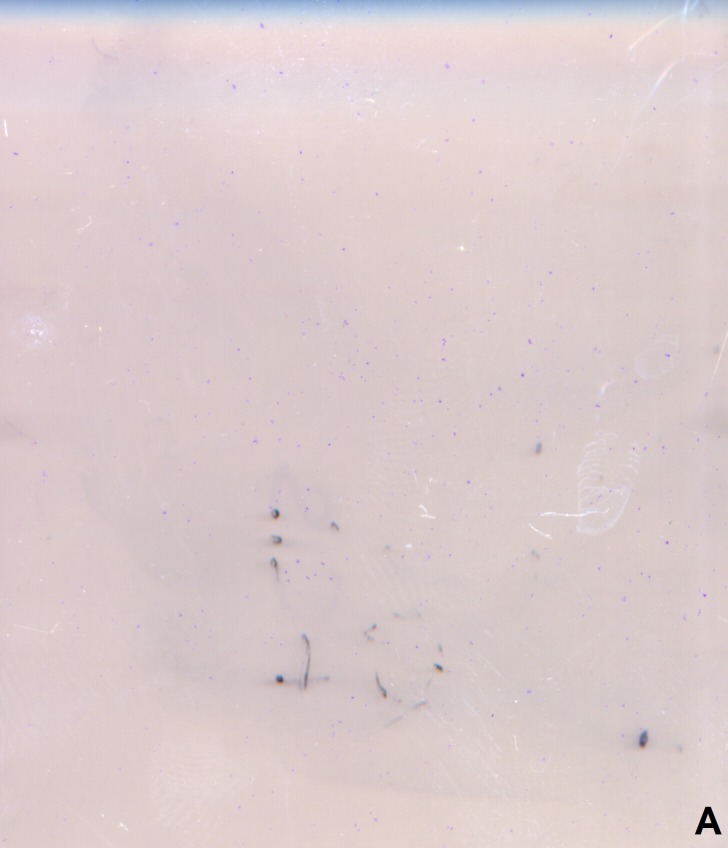

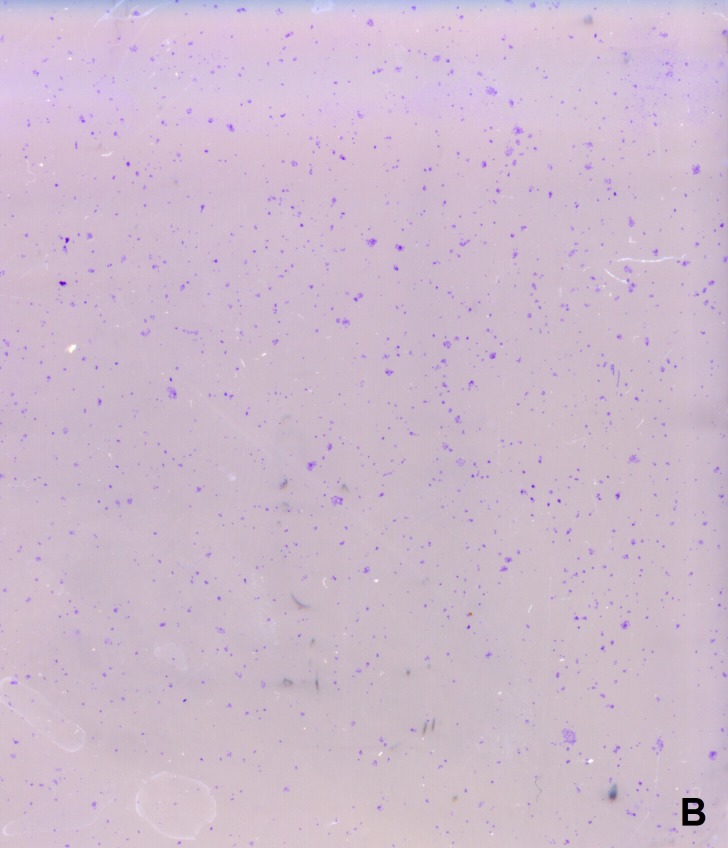

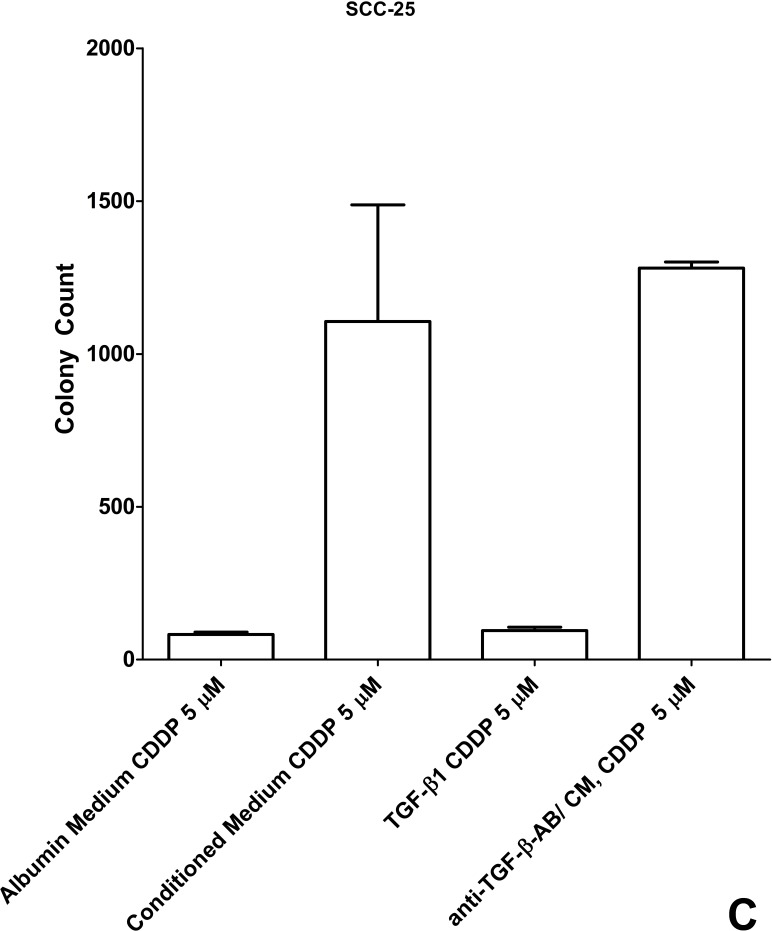

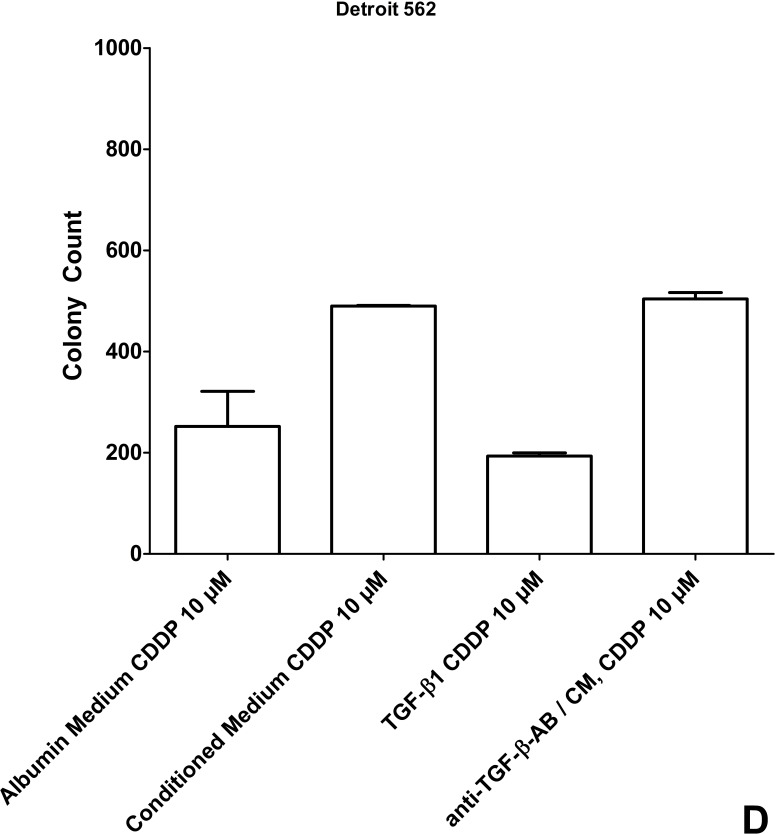
Clonogenic assay after treatment with albumin medium/conditioned medium and CDDP 0 μM, 5 μM, 10 μM **A.** Picture of the clonogenic assay of the control SCC-25 cells after treatment with 5 μM CDDP. **B.** Picture of the clonogenic assay of the SCC-25 cells cultured in co-culture conditioned medium after treatment with 5 μM CDDP. **C.** Number of colonies as a function of treatment. This demonstrates the significant increased colony forming ability of the SCC-25 cells after treatment with co-culture conditioned medium or co-culture conditioned medium/anti-TGF-β-antibody (1.5 μg/ml) and CDDP 5 μM compared to controls or cells treated with TGF-β1 1ng/ml. **D.** Number of colonies as a function of treatment. This demonstrates the significant increased colony forming ability of the Detroit 562 cells after treatment with co-culture conditioned medium or co-culture conditioned medium/anti-TGF-β-antibody (1.5 μg/ml) and CDDP 10 μM compared to the controls or cells treated with TGF-β1 1ng/ml.

Cell cultures were exposed to CDDP ranging from 0 μM to 50 μM and cell viability was assessed with MTT assays. Half-maximal inhibitory CDDP concentration was calculated using 4-parameter nonlinear logistic regression. The IC_50_ for native SCC-25 cells was 6.24 μM (95% CI 5.43 μM to 7.06 μM). Treatment with conditioned medium significantly increased CDDP-chemoresistance (Figure 3A). It more than doubled the IC_50_ of SCC-25 cells to 13.05 μM (95% CI 10.35 μM to 15.76 μM; *p* < 0.005). In addition to viability assays, clonogenic assays following exposure to 5 μM CDDP were performed in control and co-culture conditioned medium treated cells. In control cells, 5 μM CDDP reduced colony number from 3116±654 to 82±8 (91% reduction). In co-culture conditioned medium-treated cells, 5 μM CDDP reduced colony number from 1805±131 to 1106±381 (39% reduction). This means CDDP induced significant less (*p* = 0.013) reduction of colony numbers in co-culture cells than in standard-culture cells, suggesting an increased CDDP-chemoresistance following exposure to conditioned medium (Figure 4A-4C). In addition, SCC-25 cells were incubated with 1ng/ml TGF-β1 and CDDP to evaluate the specific effect of TGF-β1 on CDDP resistance (Figure 3A). The IC_50_ following TGF-β1 exposure (6.6 μM; 95% CI 5.47 μM to 7.73 μM) did not differ from the IC_50_ following standard medium (*p* > 0.2). TGF-β1 treatment did not alter clonogenity after treatment with 5 μM CDDP (95±11, *p* > 0.1, 89% reduction) compared to controls (Figure 4C).

To support the observation that TGF-β1 is not involved in co-culture conditioned medium induced chemoresistance, a neutralizing dose of anti-TGF-β (1.5 μg/ml) was added to the co-culture medium before viability and clonogenity assays were performed. Despite that, the chemoresistance mediating effect of co-culture conditioned medium was preserved. Also with anti-TGF-β, the IC_50_ of CDDP following administration of co-culture conditioned medium remained significantly higher than in control experiments (10.4 μM, 95% CI 9.2 to 11.5 μM (*p* < 0.05) (Figure 3A). The effect of co-culture conditioned medium on clonogenity after treatment with 5 μM was not altered by anti-TGF-β antibody treatment (1281±20, 13% reduction, *p* > 0.5) (Figure 4C).

### Treatment with co-culture conditioned medium increased CDDP resistance of detroit 562 cells

The same experiments were repeated in Detroit 562 cells. The IC_50_ of CDDP for native Detroit 562 cells was 13.1 μM (95% CI 9.8 to 16.5 μM). Treatment with co-culture conditioned medium more than doubled the IC_50_ to 26.8 μM (95% CI 12.7 to 41.0 μM) (p < 0.01) (Figure 3B). In control cells 10 μM CDDP reduced the colony number to 252±69 (73% reduction). In co-culture conditioned medium treated cells 10 μM CDDP reduced the colony number to 490±1 (50% reduction) (*p* < 0.05) (Figure 4D). Treatment with 1 ng/ml TGF-β1 had no influence on the IC_50_ of CDDP in Detroit 562 cells, the IC_50_ remained stable with 16.9 μM (95% CI 14.9 to 19 μM, *p* > 0.05) (Figure 3B). TGF-β1 treatment did not alter clonogenity after treatment with 10 μM CDDP (193±6, *p* > 0.3) compared to controls cells (Figure 4D). Accordingly, treatment with co-culture conditioned medium containing a neutralizing dose of anti-TGF-β1-antibody (1.5 μg/ml) did not change the IC_50_ of CDDP in Detroit 562 cells compared to co-culture conditioned medium treated Detroit 562 cells (IC_50_ 27.5 μM, 95% CI 18.5 to 36.3 μM, *p* > 0.05) (Figure 3B). Treatment with anti-TGF-β-antibody/co-culture conditioned medium and 10 μM CDDP did not alter the effect of conditioned medium on clonogenity of Detroit 562 cells (504±6, *p* > 0.05) (Figure 4D) (Table 2).

**Table 2:** Treatment with co-culture conditioned medium resulted in increased CDDP resistance (measured with cell viability and clonogenity) in both cell lines

Cell type	Parameter	Cond. Medium	TGF-β1	Cond.Medium + anti TGF-β
**SCC-25**	IC_50_	increased ***	no change	increased***
	Clonogenity after 5μM CDDP	increased**	no change	increased***
**Detroit 562**	IC_50_ in μM	increased**	no change	increased**
	Clonogenity after 10μM CDDP	increased*	no change	increased***

TGF-β1 treatment did not influence CDDP resistance. A neutralizing assay performed with anti-TGF-β antibody supported this concept because it did not alter the CDDP resistance induced by co-culture conditioned medium.

* *p* < 0.05

** *p* < 0.01

*** *p* < 0.001.

## DISCUSSION

EMT is a reversible cellular process caused by the interaction of epithelial tumor cells and tumor-associated fibroblasts. During EMT, epithelial tumor cells acquire characteristics of a mesenchymal phenotype. It is thought to be a relevant mechanism of cancer progression [5, 6]. In this study, we were interested in the effect of EMT on CDDP resistance of HNSCC tumor cells *in vitro*. Co-cultures of epithelial and mesenchymal cells are frequently used to model EMT *in vitro*. Here we used a direct fibroblast-tumor cell co-culture conditioned medium to induce EMT in the two human papilloma virus-negative HNSCC cell lines SCC-25 and Detroit 562.

### Co-culture conditioned medium induced EMT and increased cell viability

Co-culture conditioned medium upregulated vimentin expression and downregulated E-cadherin expression in SCC-25 cells. As expected for an epithelial cell line, baseline vimentin expression was low and baseline E-cadherin expression was high. Similar phenotypic changes were induced by treatment with 1 ng/ml TGF-β1. This TGF-β1 concentration has previously been measured in co-culture conditioned medium using enzyme-linked immunoassays (data not shown). TGF-β1 is considered a major factor for phenotypic changes in EMT [15].

Especially 46 kD vimentin protein levels increased in SCC-25 cells after treatment with co-culture conditioned medium and TGF-β1. 46 kD vimentin had been previously identified in epithelial cells under stress conditions [16]. On the protein level, co-culture conditioned medium and TGF-β1 slightly reduced the E-cadherin expression. The fact that EMT can be induced by cell free medium supports the concept that EMT mainly depends on paracrine signaling [8, 17, 18]. Beyond upregulation of mesenchymal and downregulation of epithelial markers, the co-culture conditioned medium increased cell viability in SCC 25 cells (*p* < 0.01). EMT-associated increase of cell viability is in line with several recent reports [4, 19].

This effect was not mediated by TGF-β1, since TGF-β1 reduced cell viability in SCC-25 cells (*p* < 0.001). It was demonstrated that Smad 4 is active in SCC-25 cells, which might enable a tumor suppressive effect of TGF-β1 [20]. Apparently, other factors than TGF-β1 are responsible for increased viability in SCC-25 cells exposed to co-culture conditioned medium and these factors are even able to override TGF-β1 induced viability reduction in SCC-25 cells. Moreover, the discrepancy of co-culture conditioned medium and TGF-β1 induced effects on EMT and cell viability implies that observed changes in cell viability are not caused by the acquisition of a mesenchymal phenotype (EMT), but rather an EMT associated effect of epithelial-mesenchymal crosstalk in the tumor microenvironment.

Despite lack of phenotypic changes, co-culture conditioned medium increased cell viability of Detroit 562 cells (*p* = 0.001), supporting the concept that EMT-induction and changes in cell viability may be caused by different factors. Consistent with the observations in SCC-25 cells, TGF-β1 had no influence on cell viability and clonogenity in Detroit 562 cells.

### Co-culture conditioned medium induced CDDP chemoresistance

The main goal of this study was to determine if EMT is associated with increased CDDP chemoresistance. Medium from an epithelial-mesenchymal co-culture more than doubled the IC_50_ of CDDP in MTT assays in both cell lines (*p* < 0.001) (Figure 2). These results are consistent with the outcome of clonogenic assays. The anti-clonogenic effect of CDDP was significantly reduced after pretreatment with co-culture conditioned medium (*p* < 0.05) (Table 2). Hazlehurst et al. observed in 2001 that tumor microenvironment is involved in cell cycle arrest and drug resistance. This so called microenvironment related drug resistance is mediated through survival pathways activated as a result of cell-cell-adhesion (cell-adhesion mediated drug resistance) and extracellular matrix derived adhesive signals, which reduce the cytotoxic effect of CDDP [21]. Niessner et al. reported that the release of paracrine signaling factors produced by carcinoma-associated fibroblasts stimulate survival pathways such as the Akt-pathway and reduce cytotoxic effects of chemotherapies [22]. We treated HNSCC cell lines with a cell-free conditioned medium, suggesting the latter being responsible for the increase in CDDP resistance in our experimental setting. Similar results have been demonstrated in lung cancer cells. In these cells treatment with fibroblast-conditioned medium increased Paclitaxel resistance by restraining Paclitaxel induced apoptosis by activation of both extracellular signal-regulated kinases (Erk) 1/2 and Akt kinase. In this study, treatment with fibroblast-conditioned medium did not alter the CDDP resistance of lung cancer cells *in vitro* [23].

### No effect of TGF-β1 on CDDP sensitivity

TGF-β1 did not increase the IC_50_ of CDDP of HNSCC cells *in vitro*. The IC_50_ of control and TGF-β1 treated cells was remarkably similar. Consistently, treatment with TGF-β1 had no influence on colony forming ability following CDDP-treatment. To support this observation, an additional experiment with co-culture conditioned medium supplemented with a neutralizing anti-TGF-β antibody was performed. Anti-TGF-β did not antagonize chemoresistance induction by co-culture conditioned medium in both cell lines. This supports the concept that increased CDDP-resistance is not caused, but merely associated with the acquisition of a mesenchymal phenotype. The factors leading to increased CDDP resistance following epithelial-mesenchymal interaction are not yet identified, but it doesn't seem to be TGF-β1. Gilbert et al. reported that IL-6 (Interleukin-6) and TIMP (Tissue Inhibitor of metalloproteinase) could be possible tumor stroma-derived factors, which promote the survival of cancer cells [24]. Other fibroblast-produced mediators which might mediate EMT-associated chemoresistance, are HGF (human hepatocyte growth factor), MED12 (mediator complex subunit 12) and PGE-2 (Prostaglandine-E2) [15].

## MATERIALS AND METHODS

### Cell lines

Human gingival fibroblasts (HGF) cells were purchased from Cell Line Service, Eppelheim, Germany [1, 25]. They were cultured in DMEM-low glucose (PAA, Pasching, Austria) supplemented with 10% foetal bovine serum (FBS) (PAA), 2 mM l-glutamine, 100 units/ml penicillin, and 100 μg/ml streptomycin. SCC-25 were purchased from German Collection of Microorganisms (DSMZ, Braunschweig, Germany) and Detroit 562 cells from the Cell Lines Service (Eppelheim, Germany), and were cultured in DMEM/F12 (PAA) supplemented with 10% FBS (PAA), 2 mM l-glutamine, 100 units/ml penicillin, and 100 μg/ml streptomycin [1].

### Co-culture conditioned medium

For the production of co-culture conditioned medium, 4 × 10^4^ /ml SCC-25 or Detroit 562 cells and 1 × 10^4^ HGF cells/ml were plated in 250 ml cell culture flasks and cultured for 72 hours in 15 ml foetal bovine serum-containing medium (1:1 mix of DMEM/F12 (PAA) and DMEM-low glucose (PAA) supplemented with 10% foetal bovine serum (FBS) (PAA), 2 mM l-glutamine, 100 units/ml penicillin, and 100 μg/ml streptomycin). Then the cells were washed twice with Dulbecco's Phosphate-Buffered Saline (DPBS) (Biowhittaker^®^, Belgium) and the serum-containing medium was replaced by 15 ml albumin-containing medium (7,5 ml DMEM/F12 (PAA) and 7,5 ml DMEM-low glucose (PAA) supplemented with bovine serum albumin (BSA, PAA) (0.4 g albumin/100 ml medium) replacing the protein content of 10% FBS, 2 mM l-glutamine, 100 units/ml penicillin, and 100 μg/ml streptomycin). Albumin-containing medium was left 48 hours on the co-culture allowing interacting epithelial cells and fibroblasts to secrete EMT-related factors into the medium. Afterwards, the co-culture conditioned medium was collected and cells were counted. The co-culture conditioned medium was portioned according to cell numbers as described by Hassona et al. [18]. The co-culture conditioned medium was sterile-filtered and stored at −80°C.

### Stimulation of SCC-25/Detroit 562 cells with co-culture conditioned medium and TGF-β1

To induce EMT in the first experimental arm, SCC-25/Detroit 562 cells were treated with 7 ml co-culture conditioned medium per 50 ml cell culture flask for 72 hours. The medium was changed daily. To assess the effects of TGF-β1 in the second experimental arm, SCC-25/Detroit 562 cells were cultivated in albumin-containing medium supplemented with TGF-β1 1 ng/ml (R&D Systems^®^, Minneapolis, US). Exposure conditions were the same as in the first experimental group, i.e., TGF-β1 supplemented medium was used over a period of 72 hours and media were changed daily. A TGF-β1 neutralizing assay was performed in the third experimental arm. SCC-25 cells and Detroit 562 were therefore treated with anti- TGF-β 1,-2,-3 antibody (R&D Systems^TM^, Biomedica, Vienna; Austria) 1.5 μg/ml medium, as neutralizing dose assayed by the provider and 7 ml co-culture conditioned medium per 50 ml cell culture flask for 72 hours. Exposure conditions were the same as in the other experimental groups. In the fourth experimental arm, standard medium was used with identical media changes. At the end of the stimulation period, cells were used for RNA extraction, protein isolation, MTT assays and clonogenic assays, respectively.

### RNA extraction, reverse transcription and real-time RT-PCR

A fraction of SCC-25/Detroit 562 cells was harvested and RNA was isolated using TRIzol^®^ reagent following the manufacturer's instructions (Ambion^®^, Life technologies^TM^, Thermo Fisher Scientific Inc., Waltham, MA, USA). RNA concentrations were determined by photometric measurements (BioPhotometer plus 6132, Eppendorf, Germany). Total RNA was reverse transcribed by M-MuLV Reverse Transcriptase (GeneON, Ludwigshafen, Germany) according to the manufacturer's instructions in a MyiQTM cycler (BIO-RAD Laboratories, Inc., US). Real-Time quantitative PCR (qPCR) of copy-DNA transcripts was performed in a MyiQTM cycler (BIO-RAD Laboratories Inc., Hercules, CA, US) using iTaqTM Universal SYBR^®^ Green Supermix (BIO-RAD Laboratories, Inc., Hercules, CA, US). β-Actin primers were purchased from Invitrogen^TM^ (Darmstadt, Germany), while E-cadherin and vimentin primers were provided by Eurofins MWG Operon, Inc. (Ebersberg, Germany). β-Actin functioned well as a housekeeping gene and did not show significant changes across the three treatment conditions. Moreover, the size of the Real-Time PCR products was confirmed by agarose gel electrophoresis analysis.

### Protein isolation and western blotting

Following treatments SCC-25 and Detroit 562 cells were washed twice with DPBS and collected with Accutase (PAA), followed by cell counting and harvesting by centrifugation at 290 g, 5 minutes, 4°C. The resulting cell pellets were resuspended in 100 μl RIPA-buffer ( 50 mM Tris HCl/pH:8.0, 2 mM EDTA, 1 mM EGTA, 1 % Triton X-100, 0.25 % sodium deoxycholate, 0.1 % sodium dodecylsulfate, 150 mM NaCl, 10 mM NaF, 1 mM PMSF) / 10^6^ cells. The cell suspension was vortexed and incubated 3-times for 15 minutes on ice, homogenized in 22G needles and centrifuged at 15000 g, 15 minutes, 4°C. The cleared supernatant was subjected to protein concentration measurement using the Pierce 660 nm protein assay (Pierce, Rochford, IL, USA) according to the instructions of the manufacturer. 15 μg protein from all samples was subsequently processed for western blot analysis published previously [17], using primary antibodies: mouse monoclonal anti-vimentin (clone VI-10) IgM (Exbio, Prague, Czech Republic) at 1:200, or mouse monoclonal anti-E-cadherin (clone 36) IgG2a (BD Transduction Laboratories, BD Austria, Vienna, Austria) at 1: 2500, or anti-GAPDH (clone 6C5) IgG1 (Santa Cruz Biotechnology, Santa Cruz, CA, USA) at 1: 200. For signal detection horseradish peroxidase coupled matched secondary antibodies and chemiluminescent substrate of Thermo Fisher Scientific (Vienna, Austria) were used in conditions suggested by the manufacturer. The chemilumescence signal was imaged by an Azure C500 documentation system (Biomedica, Vienna, Austria).

### Treatment of the cells with CDDP and IC_50_ determination

Following stimulation SCC-25/Detroit 562 cells were treated with increasing doses of CDDP (0 μM, 1 μM, 2.5 μM, 5 μM, 7.5 μM, 10 μM, 20 μM, 50 μM, 100 μM) for three days. The medium was changed daily. Cell viability was plotted against CDDP-concentration and IC_50_ was calculated employing a four-parameter nonlinear regression model [26]. For the clonogenic assays, 2 × 10^4^ cells were plated in 250ml cell culture flasks. After stimulation they were treated with 5 μM CDDP for three days. The media were changed daily.

### MTT- assay

Cell viability was evaluated by MTT-assays using the tetrazolium salt method. The MTT-assay is a quantitative colorimetric method used to determine metabolic activity [27]. After three days of CDDP treatment, 10 μl of 5 mg/ml MTT salt (in DMEM/F12 (PAA) supplemented with 10% FBS (PAA), 2 mM l-glutamine, 100 units/ml penicillin, and 100 μg/ml streptomycin) was administered to the cells (100 μl). Cells were incubated for 4h at 37°C and then the formazan reaction product was dissolved using 10% sodium dodecylsulphate in 10 mM HCl at 37°C for 12 hours. Absorbance at 550 nm was measured with a microtiter plate reader (Athos 2010, Salzburg, Austria). The MTT-tests were performed in four independent sets containing at least six biological repeats.

### Clonogenic assay

For analysis of the anti-clonogenic effect of CDDP on the tumor cells we used the modified clonogenic assay described by Phuk and coworkers [14]. SCC-25/Detroit 562 cells were washed with PBS and cultured in 250 ml tissue culture flasks in DMEM/F12 (PAA) supplemented with 10% FBS (PAA), 2 mM l-glutamine, 100 units/ml penicillin, and 100 μg/ml streptomycin for 14 days. After 14 days, cultures were fixed and stained in 0.5% gentian violet dissolved in methanol. Subsequently, the stained flasks were scanned in 1200 dpi resolution using a commercial flatbed scanner. Based on the resulting micrographs, colonies were counted and occupied areas were measured semi-automatically using a macro written in imageJ/FIJI macro language [28]. Background subtraction was performed on single images using rolling-ball-algorithm [29]. Micrographs were subjected to color deconvolution [30] and filtered using a Fourier band-pass filter. Colonies were segmented using auto-thresholding algorithms [31-35]. Segmented colonies were counted and occupied areas were measured. The clonogenic assays were performed in three independent sets.

### Data analysis

Data were presented as mean +/− standard deviation (SD) unless indicated otherwise. The IC_50_ +/− 95% confidence intervals (CI) of CDDP were calculated with four-parameter nonlinear logistic regression using CurveExpert Professional (Daniel Hyams, Hixson, TN, USA). The results of real time PCR analysis were analyzed with GraphPad Prism 4.03 (GraphPad Software Inc, San Diego, CA, USA). Mean values among groups were compared with unpaired *t*-tests. MTT-changes in co-culture conditioned medium treated cells *vs*. controls were tested with unpaired *t*-test. For evaluation of clonogenic assays, a two-factorial analysis of variance with colony number as the response parameter was used. Cell culture medium (co-culture *vs*. standard) and CCDP exposure (0 μM *vs* 5 μM or 10 μM) served as factors. The interaction term served to indicate cell-culture mediated differences in CDDP sensitivity. For this data analysis, SPSS 22 was used (IBM Corporation, Armonk, NY, USA)
